# Association between Peri-Implant Soft Tissue Health and Different Prosthetic Emergence Angles in Esthetic Areas: Digital Evaluation after 3 Years’ Function

**DOI:** 10.3390/jcm11216243

**Published:** 2022-10-23

**Authors:** Diego Lops, Eugenio Romeo, Stefano Calza, Antonino Palazzolo, Lorenzo Viviani, Stefano Salgarello, Barbara Buffoli, Magda Mensi

**Affiliations:** 1 Department of Prosthodontics, Dental Clinic, School of Dentistry, University of Milan, 20100 Milan, Italy; 2 Unit of Biostatistics and Bioinformatics, Department of Molecular and Translational Medicine, University of Brescia, 25121 Brescia, Italy; 3 Department of Surgical Specialties, Dental Clinic, School of Dentistry, University of Brescia, 25125 Brescia, Italy; 4 Section of Anatomy and Physiopathology, Department of Clinical and Experimental Sciences, University of Brescia, Viale Europa 11, 25123 Brescia, Italy

**Keywords:** dental implant, emergence angle, retrospective study, sub-crestal placement, emergence profile

## Abstract

Background: The aim of the present retrospective study was to assess peri-implant soft tissue health for implants restored with different prosthetic emergence profile angles. Methods: Patients were treated with implants supporting fixed dentures and were followed for 3 years. Buccal emergence angle (EA) measured at 3 years of follow-up visits (t1) were calculated for two different groups: Group 1 (153 implants) for restorations with angle between implant axis and prosthetic emergence angle from ≥30°, and Group 2 (67 implants) for those with angle ≤30°, respectively. Image J software was used for the measurements. Moreover, peri-implant soft tissue parameters such as pocket probing depth (PPD), plaque index (PI) and gingival index (GI) were assessed, respectively. Results: A total of 57 patients were included in the analysis and a total of 220 implants were examined. Mean (±SD) EA in Groups 1 and 2 was 46.4 ± 12.2 and 24.5 ± 4.7 degrees, respectively. After 3 years of follow-up, a PPD difference of 0.062 mm (CI_95%_ −0.041 mm; 0.164 mm) was calculated between the two groups and was not statistically significant (*p* = 0.238). Similar results were found for PI (OR = 0.78, CI_95%_ 0.31; 1.98, *p* = 0.599). Furthermore, GI scores of 2 and 3 were found for nine implants (5.9%) in Group 1, and for five implants in Group 2 (7.5%). A non-significant difference (*p* = 0.76) was found. Conclusions: Peri-implant soft-tissue health does not seem to be influenced by EA itself, when a proper emergence profile is provided for implant-supported reconstructions in anterior areas.

## 1. Introduction

A critical role in dental implant aesthetic and functional long-term prognosis is played by peri-implant soft tissues. Mucosal level stability after implant placement could be affected by soft tissue quality and quantity, type of surgical procedure [1] and prosthetic design [2]. Peri-implant soft tissue is composed of well-keratinized oral epithelium, sulcular epithelium, and junctional epithelium, as well as underlying connective tissue. The role of an adequate band of keratinized mucosa around dental implants has been widely investigated in the literature. Even then, higher values of mucosa recession and loss of attachment were correlated with inadequate width of keratinized mucosa [3,4,5]. Keratinized oral epithelium continues in the sulcular epithelium and then in the junctional epithelium; this is a non-keratinized epithelium that, due to its unique structural and functional adaptation, plays a critical role in maintaining periodontal health by forming the front line of defense against periodontal bacterial infection. Moreover, peri-implant tissue architecture may be influenced by prosthetic emergence profile (EP) design [6,7]. Emergence profile was defined as the contour of a tooth or restoration, such as the crown on a natural tooth, dental implant, or dental implant abutment, as it relates to the emergence from circumscribed soft tissues [8]. Additionally, emergence profile zones were classified [9] in order to describe their importance in peri-implant tissue shaping and fulfilling aesthetic outcomes. Among such aesthetic criteria, interproximal papilla contour, gingival margin scalloping and buccal soft tissue thickness should be considered [10]. In addition, soft tissue architecture may contribute to preventing peri-implant soft tissue inflammation, giving patients real chances to follow proper oral hygiene indications [11]. Not only has a peri-implant tissue inflammation index addressed an adequate emergence profile, but also a proper emergence angle (EA) selection [12].

**Figure 1:**
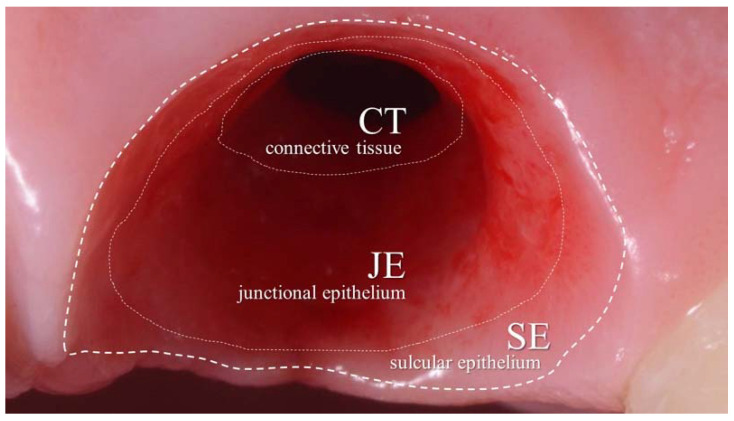
Peri-implant transition zone: connective tissue CT (1–1.5 mm) is directly connected to the peri-implant bone tissue. A junctional epithelium JE (1–2 mm) with a non-keratinized epithelium can be found above CT. A stratified squamous epithelium corresponding to the sulcular epithelium SE (1–1.5 mm) provides for the gingival margin area and is more superficial than both CT and JE, respectively.

EA was reported as the angle between the average tangent of the transitional contour relative to the long axis of a tooth [8]. It was suggested by Katafuchi et al. [12] not to overcome a 30° EA value to preserve soft tissue health in the transition zone (Figure 1).

On the other hand, in a 3-year follow-up report, no direct correlation between MBL (marginal bone level) change and emergence angles was found by Lops et al. [10]. Moreover, limited evidence about this correlation was highlighted by Mattheos et al. [13] in a critical review.

Due to the lack of agreement on this topic, more qualitative and quantitative data are needed to set further conclusions. Therefore, the primary outcome of the present report was to investigate any correlation between prosthetic emergence angles (<30° and ≥30°) and probing pocket depth (PPD) for implants placed in the anterior region. Other parameters, such as as gingival (GI) and plaque (PI) indexes in different EA groups, were considered as secondary outcomes. The authors hypothesized that with a straight-to-concave prosthetic emergence profile, EA ≥ 30° may not significantly influence peri-implant soft tissue measurements if compared to values of EA < 30°.

## 2. Materials and Methods

### 2.1. Patient Selection

The present retrospective evaluation was conducted in accordance with the fundamental principles of the Helsinki Declaration. Ethical Committee agreement (Prot. No. EC 02.04.20 REF 28/20) was obtained to complete the clinical measurement procedures mentioned below. The STROBE (Strengthening the Reporting of Observational Studies in Epidemiology, strobe-statement.org (accessed on 2 February 2014)) guideline checklist of items was followed.

Patients needing an implant-supported fixed rehabilitation in anterior areas were included; the same implant system was used (Anyridge, MegaGen Implant Co., Gyeongbuk, Korea) from 2014 to 2017; clinical parameters and EA measurements were assessed.

**Figure 2:**
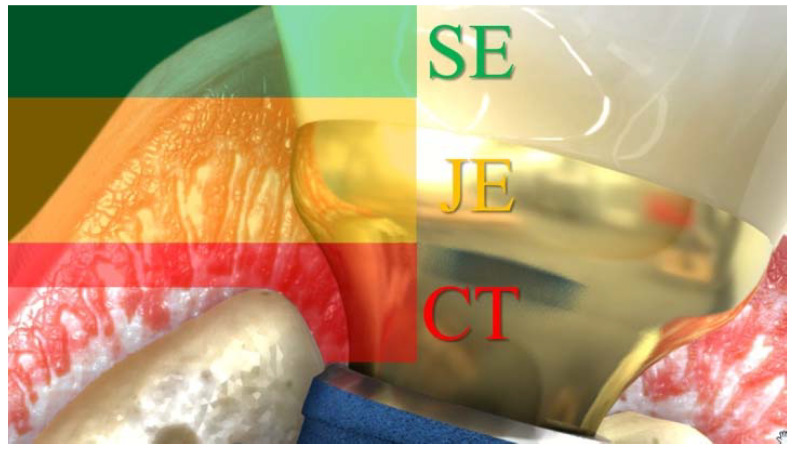
Sagittal section of the supracrestal soft tissues around implants and corresponding prosthetic components: connective tissues CT (red area), junctional epithelium JE (yellow area) and sulcular epithelium SE (green area), respectively.

Moreover, restorations with concave emergence profile (EP) at buccal aspect were included in order to not negatively interfere with all the different components of the transition zone (Figure 2), especially the biological boundary area [14]. The EP, corresponding to the restoration contour as per the definition of the Glossary of Prosthodontic Terms GTP-9 [8], was classified as concave, straight and convex on the buccal aspect during the digital EA measurement procedures.

Written consent about the study objectives was signed by the patients. Patients with single or multiple gaps were included and followed for a period of 3 years.

Patients with severe clenching or bruxism, systemic diseases, a history of radiation therapy in the head and neck region, inadequate compliance, and who were smokers (more than 15 cigarettes per day) were excluded.

The following additional data were collected: implant features as diameter and length, prosthesis type, implant site, date of prosthetic delivery.

### 2.2. Surgical and Prosthetic Procedures

As previously described by Lops et al. [10] a submerged healing technique was chosen for the implants that were placed (Anyridge, Megagen Implants, Seoul, Korea) 1 to 2 mm below the crestal level [15], as recommended by the manufacturer.

Distances of at least 3 mm, and from 1.5 to 3 mm, were chosen between implants, and between an implant and the adjacent tooth [16,17,18,19], respectively.

Only restorations from the premolar to the contralateral area were considered: fixed single crown (SC) and partial fixed prosthesis (FPD) were considered, respectively. For cemented restorations, abutments were torqued down to 25 Ncm and a temporary cement (Temp-Bond Clear, Kerr Corporation, Orange, CA, USA) was used. Differently, a torque of 25 Ncm was used to secure screw-retained prostheses.

### 2.3. Clinical and Digital Evaluations

Probing pocket depth (PPD), plaque index (PI) and gingival index (GI) [20,21,22,23] were assessed with a calibrated plastic probe (TPS probe, Vivadent, Schaan, Liechtenstein). Four sites for each implant (mesial, distal, buccal and lingual) were considered for recording probing depth scores.

GI scores ranged from 0 to 3 (0 = normal gingiva; 1 = mild inflammation: slight change in color, slight oedema. No bleeding on probing; 2 = moderate inflammation: redness, oedema and glazing. Bleeding on probing; 3 = severe inflammation: marked redness and oedema. Ulceration. Tendency to spontaneous bleeding).

Similarly, PI scores ranged from 0 to 3, respectively (0 = no plaque in the gingival area; 1 = a film of plaque adhering to the free gingival margin and adjacent area of the tooth. The plaque may only be recognized by running a probe across the tooth surface; 2 = moderate accumulation of soft deposits within the gingival pocket, on the gingival margin and/or adjacent tooth surface, which can be seen by the naked eye; 3 = abundance of soft matter within the gingival pocket and/or on the gingival margin and adjacent tooth surface).

Additionally, for GI and PI, indexes were calculated. The aforementioned parameters were recorded at 3 years of follow-up for each implant included in the present report.

**Figure 3:**
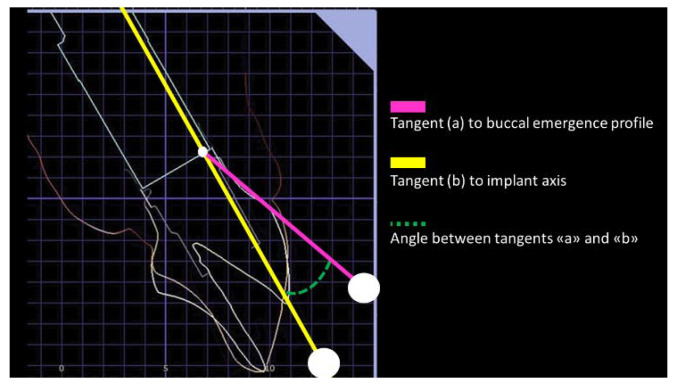
Emergence angle (EA) calculation procedure. After turning the analogic impression into a digital form, the customized emergence profile shape was planned and designed. The EA was calculated by drawing a line (yellow) parallel to the implant axis, and a pink line from the implant to the abutment connection point to the emergence profile. The angle of the intersection between pink and yellow lines resulted in the emergence angle (EA). If EA score was ≥30 degrees, the restoration was allocated to Group 1, while if it was <30 degrees the restoration was allocated to Group 2.

The angle between the tangent of the transitional contour relative to the long axis of the implant was defined as the emergence angle (EA) by following the GTP-9 indications [8]. The angle assessment was digitally performed after turning every plaster master cast into a digital form, and using the digital restoration model as a reference for the EA measurements. The buccal aspect of the restoration was used for EA calculation (Figure 3).

**Figure 4:**
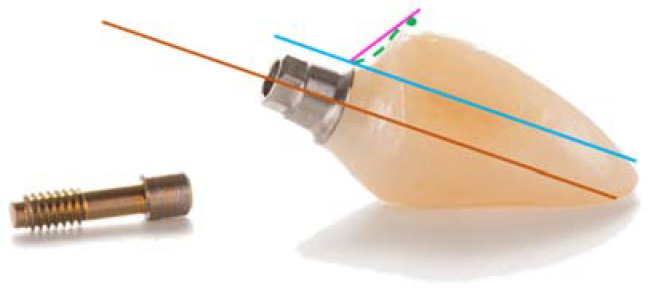
Emergence angle (EA) definition. Brown line: parallel to implant long axis. Blue line: parallel to the brown line and the line tangential to the implant shoulder. Pink line: from implant to abutment connection point to the emergence profile. The angle of the intersection between pink and blue lines resulted in the emergence angle (EA). Green line: buccal emergence profile (EP) shape. A concave area provides a support to the junctional epithelium, while the convex area supports the sulcular epithelium.

The definitive restoration EA angle was used for the group allocation (Figure 4).

**Figure 5:**
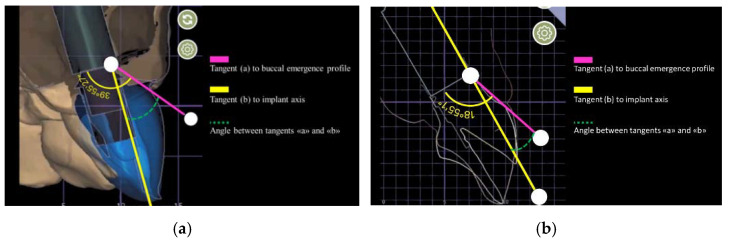
(**a**) Emergence angle (EA) calculation procedure. An EA score ≥30 degrees allocated the restoration to Group 1; (**b**) Emergence angle (EA) calculation procedure. An EA score <30 degrees allocated the restoration to Group 2.

Group 1 EA ≥ 30°, Group 2 EA < 30° (Figure 5).

The transmucosal abutment was considered as a part of restoration. The shape and emergence angle (EA) of each prosthesis was selected by the dental technician depending on the specific features of the edentulous site to be restored. Measurements were repeated twice by the same operator (LV), and intra-operator reliability was calculated.

### 2.4. Statistical Analysis

Data were collected at site level. Quantitative variables were described using mean and standard deviation while categorical variables were summarized as counts and percentages. PPD was modelled at site level using a linear mixed model (LMM), with random intercept, in order to account for within-patient data clustering. Similarly, both PI and GI (coded 0 or greater than 0), considered as binary outcomes at site level and nested within the patient, were modelled using generalized linear mixed models (GLMM) assuming a binomial family distribution. Results are reported as estimates and corresponding 95% confidence intervals. All tests were two-sided and assumed a 5% significance level. All analyses were performed using “R project” statistical computing and graphics software (version 4.2.1, https://www.r-project.org (accessed on 23 April 2022)).

## 3. Results

Fifty-seven patients (24 males and 33 females, respectively), aged from 24 to 74 years (mean age 51.2 ± 27.2 years), treated with a total of 220 implants, and followed in a 3-year period from the definitive prosthesis installation, were included in the present study. Implant length is reported in Table 1 and Table 2. Fixture distribution by implant site is reported in Table 3.

Furthermore, a descriptive analysis of gender, systemic diseases and smoking habit distribution for the different EA groups is reported in Table 4. Distribution of prosthesis type was as follows: 34 SC: single crown; 62 FPD: fixed partial denture. The mean restorations EA in Groups 1 and 2 was 46.4 ± 12.2 and 24.5 ± 4.7 degrees, respectively. A mean PPD of 1.86 ± 0.35 mm and 1.81 ± 0.33 mm were found, respectively, in Group 1 and 2 (Table 5).

A PPD difference of 0.062 mm was calculated between the two groups and was not statistically significant (*p* = 0.237). No statistical difference emerged when considering the implant site when the values of Groups 1 and 2 were compared (Table 6).

The PI index in the two groups was scored as positive in 82 and 87% of implants, respectively, for Groups 1 and 2. On the whole, 184 (84%) of the 220 sites were scored as positive after 3 years of follow-up (Table 7). The difference between the two groups was not statistically significant (*p* = 0.599). Furthermore, no statistical difference of positive values was shown considering the implant site when the values of Groups 1 and 2 were compared (Table 8). GI index in the two groups was scored as 0 in 94 and 93% of implants, respectively, for Groups 1 and 2 (Table 9). Profuse bleeding at probing was diagnosed nine (5.9%) and five times (7.5%) for Groups 1 and 2, respectively. Such difference was not statistically significant (*p* = 0.76).

## 4. Discussion

**Figure 6:**
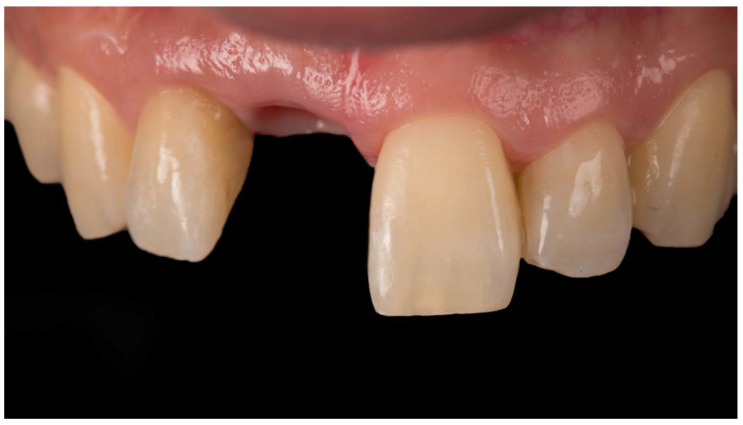
Stable peri-implant soft tissues before screwing the implant-supported restoration. Frontal view.

**Figure 7:**
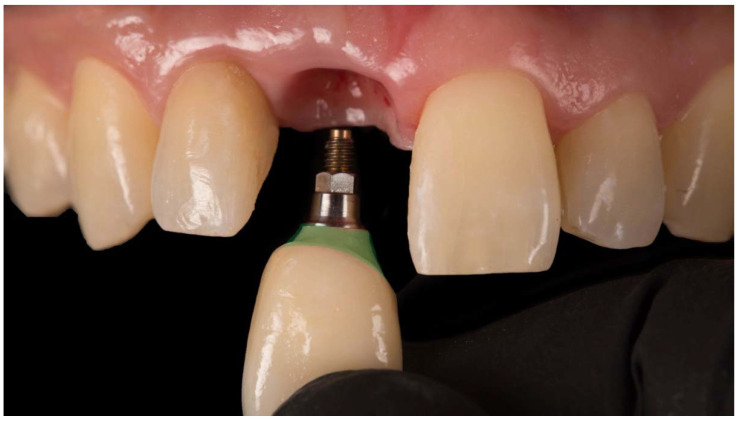
The restoration area marked in green will face peri-implant soft tissues from the junctional to the sulcular epithelium areas, respectively. Frontal view.

**Figure 8:**
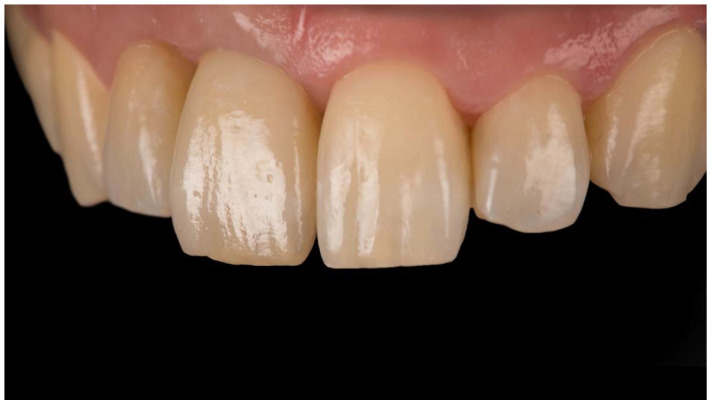
Implant-supported restoration in place.

Final implant-supported restoration contour is crucial to achieve esthetic outcome (Figure 6, Figure 7 and Figure 8). Different transition zone areas were identified with different features [14] and described as the 1 mm subgingival area apical to the free gingival margin. This so-called esthetic area should be convex in order to properly support the free gingival margin, and its shape is directly correlated to the buccal-to-palatal implant inclination. Secondly, a boundary area apical to the esthetic zone measures approximately 1–2 mm and should be concave in order to leave proper space for the soft tissues. The implant position and the choice of the restoration prosthetic components may interfere with the soft tissue thickness and the stability of apical-to-coronal transition zone dimensions. More apical and directly coronal to the implant-to-abutment connection area is 1–1.5 mm of connective tissue related to the peri-implant bone stability. The vertical dimension of such space is dependent on the implant design and the crestal or sub-crestal implant placement.

Even though such transition zone areas are actually well known and the geometry of prosthetic restoration is accepted, there is no clear quantitative measurement of the parameters related to a proper prosthetic profile contour. The emergence profile and angle concepts were used to describe of such circumscribed soft tissues. As reported by the ninth edition of the Glossary of Prosthodontic Terms [8], the “emergence profile” (EP) and “emergence angle” (EA) are described similarly for both natural teeth and implant prostheses; however, extrapolating these terms on implant prostheses, EP is defined as the restoration contour, including the abutment and crown complex. Differently, EA is defined as the angle of an implant restoration transitional contour as determined by the relation of the surface of the abutment to the long axis of the implant body.

In the present study, ≥30 and <30° EA were investigated in two groups of implant-supported reconstructions, respectively. All implants were restored by means of a 5° internal conical connection and a platform shifting of the prosthetic abutments from the fixture diameter. Such feature, related to the implant-to-abutment connection, seems to be effective in the maintenance of peri-implant bone stability in the mid-to-long term [10]. After 3 years of follow-up, EA digitally measured on the buccal side was correlated to peri-implant soft tissue parameters as PPD, PI and GI indexes, respectively. No significant differences between the two groups were found for each parameter investigated. Such finding shows that the EA parameter may not itself affect peri-implant soft tissue stability, but only if related to a specific emergence profile shape; in a cross-sectional study by Yi et al., the influence of prosthetic features was investigated through a comprehensive analysis with other known risk factors. EA had a significant effect on the prevalence of peri-implantitis only if associated with a straight or convex EP [24]. On the contrary, if it was associated with a concave emergence profile, EA was not related to an increased peri-implantitis rate. These outcomes perfectly agree with those of the present paper, with EA measured at the buccal aspect of the transition zone. A similar conclusion was also reported by other authors when EA was measured at the inter-proximal aspect: in a cross-sectional radiographic analysis by Katafuchi et al. [12], the highest peri-implantitis rate (37.8%) was observed only if the restoration emergence was combined with a convex profile. Similar outcomes were found in a retrospective analysis by Lops et al. [10]: marginal bone loss and plaque indexes were not statistically different with interproximal EA > and ≤30 degrees after 3 years of follow-up. The EP in all the restorations were straight or concave. Even in the similar conclusions by Katafuchi et al. [12] and Lops et al. [10], a different method was used to assess EA parameters in the present report; in fact, not a radiographic but a digital workflow was followed to investigate buccal EA.

From a clinical point of view, the present study outcomes may lead to the conclusion that EA > 30 degrees can be chosen to plan implant-supported reconstructions with high esthetic impact without an increase of peri-implant disease risk, as long as a concave EP and a stable implant-to-abutment connection is provided. Even then, access to oral hygiene procedures should be guaranteed to avoid the risk of peri-implant disease [13,24,25,26] by avoiding prosthesis buccal over-contouring in the esthetic area. Nevertheless, more prospective and long-term data are required to confirm this trend.

## 5. Conclusions

Peri-implant soft-tissue stability does not seem to be influenced by EA itself when a correct emergence profile is provided for implant supported reconstructions in anterior areas, even if this parameter is more than 30 degrees.

## Figures and Tables

**Table 1:** Distribution of implant length in Group 1 (EA ≥ 30°) and Group 2 (EA < 30°).

		Group 1	Group 2	Total
**Implant** **Length (mm)**	7	3 (2%)	1 (1.5%)	4 (2%)
	8.5	4 (3%)	1 (1.5%)	5 (2%)
	10	20 (13%)	6 (9%)	26 (12%)
	11.5	2 (1%)	8 (12%)	10 (5%)
	13	100 (65%)	41 (61%)	141 (64%)
	15	24 (16%)	10 (15%)	34 (15%)
Total		153	67	220

**Table 2:** Frequency of implant diameter in Group 1 (EA ≥ 30°) and Group 2 (EA < 30°).

		Group 1	Group 2	Total
**Implant** **Diameter (mm)**	3.5	38 (25%)	25 (37%)	63 (29%)
	4.0	69 (45%)	23 (34%)	92 (42%)
	4.5	43 (28%)	19 (28%)	62 (28%)
	5.0	1 (0.6%)	0 (0%)	1 (0.4%)
	5.5	1 (0.6%)	0 (0%)	1 (0.4%)
	6.5	1 (0.6%)	0 (0%)	1 (0.4%)
Total		153	67	220

**Table 3:** Frequency of implant distribution by implant site in Group 1 and Group 2.

		Group 1	Group 2	Total
**Implant Placement region**	Incisor and Canine	56 (37%)	36 (54%)	92 (42%)
	Premolar	97 (63%)	31 (46%)	128 (58%)
Total		153	67	220
Upper or Lower Jaw	Mandible	98 (64%)	30 (45%)	128 (58%)
	Maxilla	55 (36%)	37 (55%)	92 (42%)
Total		153	67	220

**Table 4:** Gender, systemic diseases and smoking habit distribution of Group 1 (EA ≥ 30°) and Group 2 (EA < 30°) patients.

		Group 1	Group 2	Total
**Gender**	F	82 (54%)	43 (64%)	125 (57%)
	M	71 (46%)	24 (36%)	95 (43%)
Total		153	67	220
Systemic diseases & smoking habit	Diabetes	12 (8%)	6 (9%)	18 (8%)
	Bisphosphonate ex-consumers	1 (0.6%)	0 (0%)	1 (0.4%)
	Smokers	35 (23%)	10 (15%)	45 (20%)
	Non-smokers and no systemic diseases	105 (69%)	51 (76%)	156 (71%)
Total		153	67	220

**Table 5:** Probing pocket depth in Group 1 (EA ≥ 30°) and Group 2 (EA < 30°).

		Group 1	Group 2	Overall
**PPD**	Mean (SD)	1.86 (0.35)	1.81 (0.33)	1.85 (0.34)
	Median (IQR)	2.00 (0.33)	2.00 (0.33)	2.00 (0.33)

**Table 6:** *Linear mixed models* for PPD parameter.

Group Comparison		Sup/Inf	Ant/Post	Difference	Lower.CL	Upper.CL	*p* Value
**Group 1**	Group 2	Sup	Ant	0.166	−0.010	0.342	0.06484986
Group 1	Group 2	Inf	Ant	0.068	−0.115	0.252	0.46485740
Group 1	Group 2	Sup	Post	0.078	−0.103	0.260	0.39443006
Group 1	Group 2	Inf	Post	−0.019	−0.193	0.155	0.82867473
**Group Comparison**				**Difference**	**Lower.CL**	**Upper.CL**	***p*** **Value**
**Group 1** **(≥30°)**		Group 2 (<30°)		0.062	−0.041	0.164	0.2379016

**Table 7:** Plaque index in Group 1 (EA ≥ 30°) and Group 2 (EA < 30°).

		Group 1	Group 2	Total
**PI**	Positive (from 1 to 3)	126 (82%)	58 (87%)	184 (84%)
	Negative (0)	27 (18%)	9 (13%)	36 (16%)
Total		153	67	220

**Table 8:** *Linear mixed models* for PI parameter.

Group Comparison		Sup/Inf	Ant/Post	OR	Asymp.LCL	Asymp.UCL	*p* Value
**Group 1**	Group 2	Sup	Ant	1.273	0.269	6.024	0.7610423
Group 1	Group 2	Inf	Ant	0.803	0.162	3.976	0.7884486
Group 1	Group 2	Sup	Post	0.707	0.121	4.143	0.7003673
Group 1	Group 2	Inf	Post	0.446	0.083	2.395	0.3463866
**Group Comparison**				**OR**	**Asymp.LCL**	**Asymp.UCL**	***p*** **Value**
**Group 1** **(≥30°)**		Group 2 (<30°)		0.778	0.305	1.984	0.5991774

**Table 9:** Gingival index in Group 1 (EA ≥ 30°) and Group 2 (EA < 30°).

		Group 1	Group 2	Total
GI	0	144 (94%)	62 (93%)	206 (94%)
	1	0 (0%)	0 (0%)	0 (0%)
	2	5 (3%)	2 (3%)	7 (3%)
	3	4 (3%)	3 (4%)	7 (3%)
Total		153	67	220

## Data Availability

The data presented in this study are available on request from the corresponding author. The data are not publicly available due to privacy policies.

## References

[1] Linkevicius T., Puisys A. (2015). Crestal bone stability around implants with horizontally matching connection after soft tissue thickening: A prospective clinical trial. Clin. Implant Dent. Relat. Res..

[2] Kim S., Oh K. (2010). Influence of transmucosal designs of three one-piece implant systems on early tissue responses: A histometric study in beagle dogs. Int. J. Oral. Maxillofac. Implant..

[3] Kaddas C., Papamanoli E. (2022). Etiology and Treatment of Peri-Implant Soft Tissue Dehiscences: A Narrative Review. Dent. J..

[4] Lin G., Chan H. (2013). The significance of keratinized mucosa on implant health: A systematic review. J. Periodontol..

[5] Cinquini C., Marchio V. (2022). Histologic Evaluation of Soft Tissues around Dental Implant Abutments: A Narrative Review. Materials.

[6] Schoenbaum T.R. (2015). Abutment emergence profile and its effect on Peri-implant tissues. Compend Contin Educ. Dent..

[7] Gonzalez-Martin O., Lee E. (2020). Contour Management of Implant Restorations for optimal emergence profiles: Guidelines for immediate and delayed provisional restorations. Int. J. Periodontics Restor. Dent..

[8] Ferro K.J., Morgano S.M., Driscoll C.F., Freilich M.A., Guckes A.D., Knoernschild K.L., Twain M. (2017). The glossary of prosthodontic terms: Ninth edition. J. Prosthet. Dent..

[9] Su H., Gonzalez-Martin O. (2010). Considerations of implant abutment and crown contour: Critical contour and subcritical contour. Int. J. Periodontics Restor. Dent..

[10] Lops D., Romeo E. (2022). Marginal bone maintenance and different prosthetic emergence angles. A 3-years prospective study. J. Clin. Med..

[11] Sanz M., Lang N.P. (2011). Seventh European Workshop on Periodontology of the European Academy of Periodontology at the Parador at la Granja, Segovia, Spain. J. Clin. Periodontol..

[12] Katafuchi M., Weinstein B.F. (2018). Restoration contour is a risk indicator for peri-implantitis: A cross-sectional radiographic analysis. J. Clin. Periodontol..

[13] Mattheos N., Janda M. (2021). Impact of design elements of the implant supracrestal complex (ISC) on the risk of peri- implant mucositis and peri-implantitis: A critical review. Clin. Oral. Implant. Res..

[14] Gomez-Meda R., Esquivel J. (2021). The esthetic biological contour concept for implant restoration emergence profile design. Int. J. Esthet. Restor. Dent..

[15] Lops D., Stocchero M. (2020). Degree Internal Conical Connection and Marginal Bone Stability around Subcrestal Implants: A Retrospective Analysis. Materials.

[16] Cosyn J., Eghbali A. (2011). Immediate single-tooth implants in the anterior maxilla: 3-year results of a case series on hard and soft tissue response and aesthetics. J. Clin. Periodontol..

[17] Galindo-Moreno P., Fernández-Jiménez A. (2015). Influence of the crown-implant connection on the preservation of peri-implant bone: A retrospective multifactorial analysis. J. Oral. Maxillofacc. Surg..

[18] Lops D., Chiapasco M. (2008). Incidence of inter-proximal papilla between a tooth and an adjacent immediate implant placed into a fresh extraction socket: 1-year prospective study. Clin. Oral. Implant. Res..

[19] Lops D., Parpaiola A. (2017). Interproximal Papilla Stability Around CAD/CAM and Stock Abutments in Anterior Regions: A 2-Year Prospective Multicenter Cohort Study. Int. J. Periodontics Restor. Dent..

[20] McClanahan S., Bartizek R. (2001). Identification and consequences of distinct Löe-Silness gingival index examiner styles for the clinical assessment of gingivitis. J. Periodontol..

[21] Löe H. (1967). The Gingival Index, the Plaque Index and the Retention Index Systems. J. Periodontol..

[22] Mombelli A., Lang N.P. (1994). Clinical parameters for evalutation of dental implants. Periodontol 2000.

[23] Mombelli A., Lang N.P. (1998). The diagnosis and treatment of peri-implantitis. Periodontol 2000.

[24] Yi Y., Koo K. (2020). Association of prosthetic features and peri-implantitis: A cross sectional study. J. Clin. Periodontol..

[25] Serino G., Ström C. (2009). Peri-implantitis in partially edentulous patients: Association with inadequate plaque control. Clin. Oral. Implant. Res..

[26] De Tapia B., Mozas C. (2019). Adjunctive effect of modifying the implant-supported prosthesis in the treatment of peri-implant peri-implant mucositis. J. Clin. Periodontol..

